# Comparative 3D analyses and palaeoecology of giant early amphibians (Temnospondyli: Stereospondyli)

**DOI:** 10.1038/srep30387

**Published:** 2016-07-26

**Authors:** Josep Fortuny, Jordi Marcé-Nogué, J.-Sébastien Steyer, Soledad de Esteban-Trivigno, Eudald Mujal, Lluís Gil

**Affiliations:** 1 Institut Català de Paleontologia M. Crusafont. Z building, Universitat Autònoma de Barcelona, CP: 08193 Cerdanyola del Vallès, Barcelona, Spain; 2 Centre de Recherches en Paléobiodiversité et Paléoenvironnements, UMR 7202 CNRS-MNHN-UPMC, Muséum national d’Histoire naturelle, Bâtiment de Paléontologie, CP38, 8 rue Buffon, 75005 Paris, France; 3 Centrum für Naturkunde, University of Hamburg, CP: 20146 Hamburg, Germany; 4 Universitat Politècnica de Catalunya–BarcelonaTech, CP: 08222 Terrassa, Spain; 5 Transmitting Science, CP: 08784 Piera, Spain; 6 Departament de Geologia, Universitat Autònoma de Barcelona, CP: 08193 Cerdanyola del Vallès, Barcelona, Spain

## Abstract

Macroevolutionary, palaeoecological and biomechanical analyses in deep time offer the possibility to decipher the structural constraints, ecomorphological patterns and evolutionary history of extinct groups. Here, 3D comparative biomechanical analyses of the extinct giant early amphibian group of stereospondyls together with living lissamphibians and crocodiles, shows that: i) stereospondyls had peculiar palaeoecological niches with proper bites and stress patterns very different than those of giant salamanders and crocodiles; ii) their extinction may be correlated with the appearance of neosuchians, which display morphofunctional innovations. Stereospondyls weathered the end-Permian mass extinction, re-radiated, acquired gigantic sizes and dominated (semi) aquatic ecosystems during the Triassic. Because these ecosystems are today occupied by crocodilians, and stereospondyls are extinct amphibians, their palaeobiology is a matter of an intensive debate: stereospondyls were *a priori* compared with putative living analogous such as giant salamanders and/or crocodilians and our new results try to close this debate.

Stereospondyls are the most successful clade of temnospondyls which quickly radiated after the end-Permian mass extinction and acquired gigantic sizes (1–6 meters long) during the Triassic^1^. These non-amniotic tetrapods were secondarily (semi) aquatic, carnivorous, and very abundant in freshwater, coastal and even marine environments. Morphologically, they are characterized by a flattened skull with raised orbits, a small or absent hyobranchial apparatus but a well-developed musculature (i.e., very elongated anteriorly adductor and large depressor) and labyrinthodont teeth^2^,^3^,^4^. These characters, particularly dentition, possibly played a role in the feeding strategy: stereospondyls had conical teeth with folded dentine (plicidentine) and enamel, as well as large fangs that probably helped to catch preys^5^. This pattern is different than the thecodont dentition of crocodiles but also from the pedicellate teeth found in living salamanders.

Stereospondyls were a near-ubiquitous component of the (semi) aquatic ecosystems and the second most important group, after therapsids, in terms of Triassic biodiversity and abundance^6^,^7^. Nonetheless, their palaeobiology is still much debated: they have been historically disputed as bottom dwellers, ambushers or active predators^3^,^5^,^8^,^9^,^10^,^11^,^12^,^13^,^14^,^15^. Clades as rhytidosteids or derwentiids are considered as small predators in rivers^13^, while all further advanced stereospondyls are inferred as active swimmers in rivers, ambushers living in a broad range of habitats, or active predators present in brackish swamps, estuarines and large river deltas^13^. Stereospondyls are *a priori* compared either with living giant salamanders based on their amphibian systematics or with living crocodilians based on their analogous morphology^2^,^14^. To shed light on this debate, two well-known stereospondyls (*Edingerella madagascariensis* and *Stanocephalosaurus birdi*) were analysed. These two taxa have been chosen because they represent medium and large-sized taxa from the Early and Middle Triassic of Madagascar and North America respectively, and their cranial general shape is of particular interest to analyse the potential convergence with crocodiles. Their ecological position(s) has therefore been tested for the first time by comparing them to the living crocodile *Alligator mississipiensis* and the giant salamander *Dicamptodon ensatus*, and by using 3D Finite Element models. Adult skulls of *Stanocephalosaurus* and *Edingerella* were 3D modeled simulating three different biomechanical behaviours: bilateral and unilateral biting/prehension and a lateral biting/prehension, simulating a rapid sideways sweep of the head (see Methods, Table 1). Their skull models were scaled and compared with the two living models of *Dicamptodon* and *Alligator*. A Principal Component Analysis (PCA) was used to analyse the response of the stress levels in a multivariate framework using as variables the stress values recorded at different homologous points in each model.

Overall, the aim of this study is i) to understand the ecological position, biomechanical capabilities, phylogenetical and structural constraints of Triassic stereospondyls, and ii) to decipher their evolutionary history compared with modern taxa.

## Results

The estimated bite force that acted on the prey is recorded for the bilateral and unilateral cases as the force reaction that appears during the bite (Supplementary Table S1). The stresses distributions and values are recorded for each skull in order to compare their behaviour under the effect of the loads and constraints as defined for the different cases (Table 1). Numerical values of stress are recorded in different locations (Supplementary Tables S2–S4). Results for the Triassic tetrapods are below presented, while the results for the extant taxa are described in the Supplementary Data.

### Triassic temnospondyls

The analyses of *Edingerella madagascariensis* and *Stanocephalosaurus birdi* reveal similar patterns, but the stress levels in *S. birdi* are slightly higher than those of *E. madagascariensis*. These differences are probably due to the cranial morphology of the two taxa: *S. birdi* has more elongated preorbital region and choanae, and a slender cultriform process than *E. madagascariensis*^14^,^16^,^17^.

**Figure 1:**
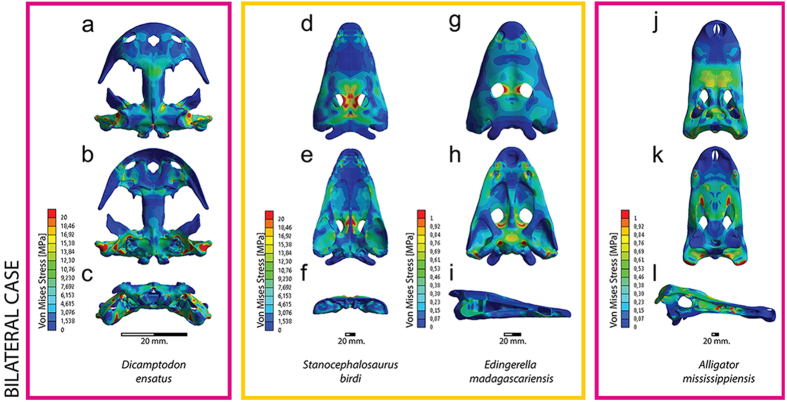
Von Mises stress results in MPa of bilateral biting in (**a–c**) *Dicamptodon ensatus*, (**d–f**) *Stanocephalosaurus birdi*, (**g–i**) *Edingerella madagascariensis*, (**j–l**) *Alligator mississippiensis. S. birdi* scaled under *D. ensatus* reference model and *E. madagascariensis* under *A. mississippiensis* reference model.

When a bilateral biting is simulated and scaled using the lissamphibian and the crocodilian parameters in *E. madagascariensis* and *S. birdi* (Fig. 1, Supplementary Video S1), the maximum stress peaks around the orbits, interorbital region, mainly around the frontals and postfrontals and surrounding bones. The main difference between the two scaled results refers to the levels of stress: the lissamphibian scaled results present the highest level of stress, while the crocodilian scaled results are the lowest ones, revealing that, from the same feeding behaviour but different structural conditions for the Triassic stereospondyls, the lissamphibian scaling has weaker capabilities in front of the rest of the crocodilian scaling or the non-scaled results. Otherwise, the general stress in both scaled cases reveals similar patterns: moderate stress levels present around the maxilla and nasals, as well as in the parietals.The stress increases around the orbits and in the posterior part of the skull (Fig. 1), excluding the tabulars, posterior part of the squamosal and quadratojugal. Similar stress patterns are observed in the palate, with moderate levels of stress around the vomerine plate, posterior ramus of the pterygoid, parasphenoid, exoccipitals and occipital condyles, while no stress is present in the cultriform process, anterior ramus of the pterygoids, quadrates and quadratojugals.

Considering the same bilateral bite under non scaled models (see Methods, Supplementary Fig. S1), the Triassic temnospondyls reveal two important regions of stress: the main area included the maxilla-nasal bones and the vomerine region, while the parietal-postparietal bones and the occipital condyles region also show moderate-high stress levels. Noteworthy, in *E. madagascariensis*, the interorbital region also has moderate-high stress levels, while in *S. birdi*, the interorbital region has low stress levels due to its more elongated skull, causing a stress belt around the prefrontals and frontals not found in *Edingerella* (Supplementary Fig. S1). The maxilla and especially the central part of the nasals present important stresses while this stress is also reflected on the anterior part of the vomerine plate. Low levels of stress are present on the preorbital-narial region, increasing around the orbits.

**Figure 2:**
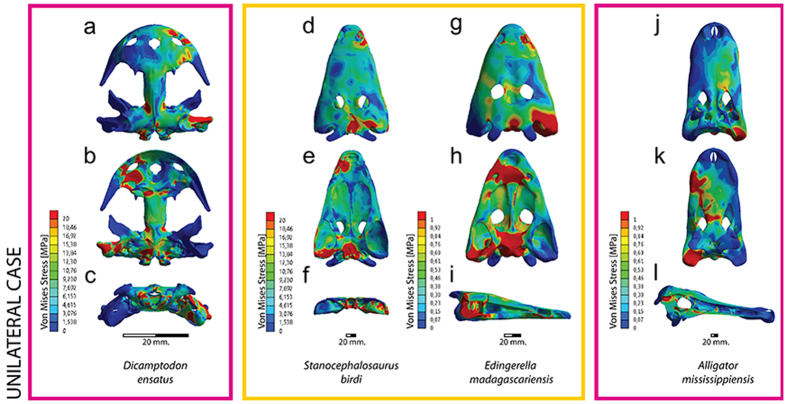
Von Mises stress results in MPa of unilateral biting. (**a–c**) *Dicamptodon ensatus*; (**d–f**) *Stanocephalosaurus birdi*; (**g–i**) *Edingerella madagascariensis*; (**j–l**) *Alligator mississippiensis. S. birdi* scaled under *D. ensatus* reference model and *E. madagascariensis* under *A. mississippiensis* reference model.

The postorbital region has moderate levels of stress. On the contrary, the tabulars, the posterior part of the squamosal and the quadratojugals present very low levels of stress. In palatal view, moderate levels of stress are present on the anterior ramus of the pterygoids and parasphenoids, and especially on the exoccipitals and occipital condyles. Regarding the unilateral bite in both temnospondyls, when the unilateral case is scaled using the lissamphibian and crocodilian conditions (Fig. 2, Supplementary Video S2), stresses highly increase around the maxilla-vomerine plate and at the quadratojugal-squamosal-supratemporal-postparietal and parasphenoid-exoccipital regions. For the rest of the skull, moderate-low levels are mainly present on the interorbital and cheek regions, while the cultriform process of the parasphenoid presents very low levels of stress. On the other hand, under this unilateral biting and non-scaled models (see Methods, Supplementary Fig. S2), the stress levels recorded are slightly higher in comparison with the bilateral loading. In both taxa, the stress patterns are extremely similar to those found in the scaled cases.

**Figure 3:**
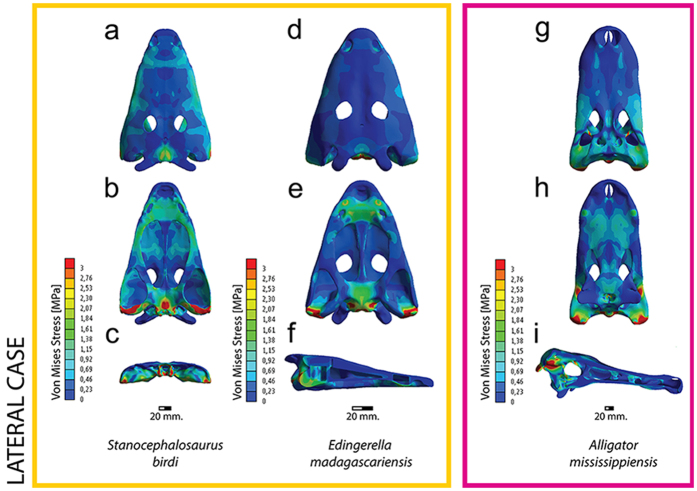
Von Mises stress results in MPa of lateral biting. (**a–c**) *Stanocephalosaurus birdi*; (**d–f**) *Edingerella madagascariensis*; (**g–i**) *Alligator mississippiensis*. Both Triassic temnospondyls scaled under *A. mississippiensis* reference model.

Under a lateral strike of the skull, the analysis reveals that under both lissamphibian and crocodilian references (Fig. 3, Supplementary Video S3), the highest stress levels peak around the parasphenoid-exoccipital and quadrate regions, with moderate levels in the whole vomerine plate. Low stresses are present along the margin of the skull roof (jugals) and postorbital. The main difference between the lissamphibian and crocodilian model reference is on the level of stress; under a crocodilian scaled loading, the stress levels (especially on the vomerine plate and parasphenoid) are lower compared to the lissamphibian scaled and the non-scaled results (Supplementary Fig. S3), while the levels of stress are intermediate between the crocodilian and the non-scaled results. In the case of the non-scaled models, the stress distribution is very similar to the scaled ones.

### Multivariate analysis

**Figure 4:**
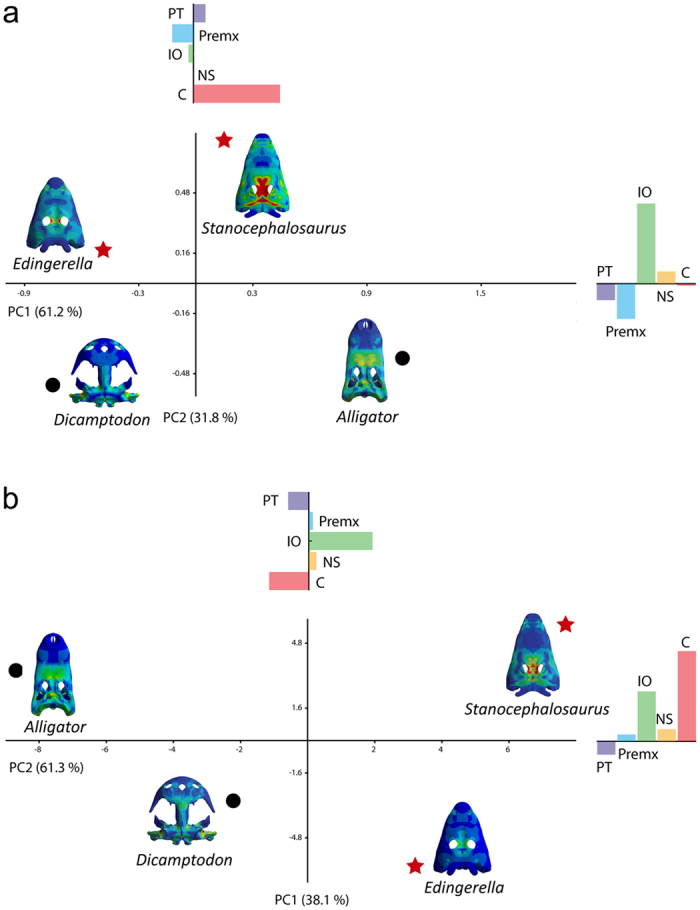
First two Principal Components of PCAs for bilateral case. (**a**) Scaled under *A. mississippiensis* reference model; (**b**) scaled under *D. ensatus* reference model. The bar graphs represent the loadings of the variables for each PC. Although all are represented at the same scale, for visualization purposes they have a different scale than the PCs dispersion graph. Percent of variance explained by each PC between brackets. Black circles correspond to the living taxa and red stars to extinct taxa. Abbreviations: C: most external point of the occipital condyle, Pt: centre of the posterior margin of the pterygoid, Premx: most anterior point between the premaxillas, IO: centre of the interorbital region, NS: middle point between the nasal sutures.

A Principal Component Analysis (PCA) has been made for bilateral biting scaled under *Alligator* and *Dicamptodon* reference models in order to calculate the distribution of the variance. The result is that the variance distribution is similar (Fig. 4). In both cases, the first principal component (PC1) explains 61% of the variance, while the second (PC2) explains more than 30% of the variance. However, the loadings of the variables for each analysis are different (Supplementary Table S5). In the PCA based on the variables scaled under the *Alligator* reference model, on its positive extreme PC1 shows high loading for the stress at the condyle. PC2 shows that the stress level in the interorbital region is very important on its positive side (Fig. 4, Supplementary Table S5). On the other hand, when the models are scaled under the *Dicamptodon* reference, PC1 shows a high stress on the interorbital region on the positive side and high stress on the condyle on the negative side (Supplementary Fig. S4). Regarding the unilateral case, more than 95% of the variance is explained by PC1, therefore no other PCs are analysed (Supplementary Table S5). The same pattern is found under both reference models, showing that the positive side of PC1 represents a huge stress value at the condyles. Lastly, considering the lateral case (excluding *D. ensatus*), the results shows the same pattern than the unilateral case (Supplementary Fig. S4, Table S5), as the condyles overcome most of the variance in the sample.

## Discussion

The palaeoecology of temnospondyls is very discussed, particularly depending on the group and the methodology used (geometric morphometrics, histology, sedimentology, palaeoenvironmental reconstruction, etc.^5^,^13^,^18^,^19^,^20^). This is particularly the case of the stereospondyls, which are *a priori* considered as convergent with living crocodilians and/or giant lissamphibians. The results of the Finite Element Analysis reveal however that these giant stereospondyls have a peculiar ecological position, which differs from those of giant salamanders and crocodiles. In this sense, FEA and PCA results demonstrate that considering the amphibian nature of the Triassic giant temnospondyls, these animals occupied a different ecological niche than living giant salamanders or crocodiles (Fig. 4b). The phylogenetical and structural constraints calculated for the giant stereospondyls allow us to precise their evolutionary history. Our results also concern (a) their cranial structural pattern, (b) dentition, and (c) evolutionary ecology.

(a) The cranial structural pattern of the studied taxa has an important role in the stress distribution pattern: the flat and broad skull of *Dicamptodon* has no stabilizing bony bridge between the maxilla and squamosal/quadrate, causing high stress distributions under different bite positions^21^. Its stress distribution pattern clearly differs from that of the large, flat and triangular skulls of the studied stereospondyls. Our results also confirm that these Triassic stereospondyls undergo a large amount of stress in the posterior part of the skull, particularly in the circumorbital and braincase regions (PCA, Fig. 4). On the contrary, the different cranial structures and the secondary palate of *Alligator* undergo less stress in the posterior cranial region. The amphibian (i.e., anamniotic) cranial structure is therefore considered as less adapted than the archosaurian/romorph cranial structure to the same loadings. It could explain the decline of the giant anamniotes from the Late Triassic, correlated with the rise of the crown archosaurs (particularly pseudosuchians) in the same aquatic ecosystems. We also confirmed that the cranial suture pattern plays an important role in the stress absorption, and in the flexion and distribution of the stress, as suggested by previous studies^15^,^21^,^22^. The role of each suture depends on its position but also on its suture type (at least eight suture types were described in temnospondyls and other tetrapods such as archosaurs^23^,^24^). In archosaurs, peculiar structures such as sinuses and inner cavities have different biomechanical implications for the stress patterns^25^. Nonetheless, the Triassic stereospondyls do not show sinuses but a narrow air cavity between the vomerine plate and the skull roof that continues through the cultriform process of the parasphenoid. This cavity is particularly interesting, especially in the vomerine region, because it spreads and reduces the stress within the skull. However, the absence of a secondary palate avoids a general stress reduction, at least during unilateral biting, as present in neosuchians^26^. The results of our FEA demonstrate that the giant stereospondyls had a powerful direct bite, as in crocodilians, and that this bite is stronger than the one of the studied giant salamanders. These stereospondyls may therefore prefer to use their powerful bite rather than their suction feeding system^5^ to catch preys. However, because of the high stress levels under this behaviour in the parasphenoid and braincase regions, these stereospondyls may not catch preys by rapid sideway sweeps of the head during active swimming, as crocodiles.

Our FEA and PCA also reinforce the importance of the occipital condyles during the different biomechanical behaviours: the stereospondyls present two occipital condyles (archosaurs have one) where the stress is concentrated in different cases (Fig. 4), and scapular elements (interclavicle and clavicles) close to the posterior part of the skull. This may limit rapid lateral movements of the head. The fact that the scapular elements are close to the skull implies that the clavicles play a key function in the skull-raising because the cleidomastoideus muscle is inserted from the dorsal process of the clavicle to the tabular horns^10^. This may explain why the tabular horns and the nearing otic notch region are generally well developed in stereospondyls, except in trematosaurians: in this group, the clavicles and the interclavicle are narrow and slender^27^ and located more posteriorly, and the tabular horns and otic notch region less developed. This suggests a lower skull raising function but more rapid lateral movements of the head under an active swimming for the trematosaurians.

(b) Regarding the dentition, early amphibians generally possess haplodont, unicuspid and conical teeth, with large bases and subthecodont attachment^28^. In particular, temnospondyls are characterized by labyrinthodont teeth with an intricated plicidentine and enamel^29^. Tooth rows are present on the maxilla-ectopterygoid and vomerine, while in some clades such as stereospondyls, large fangs probably helped to catch preys^5^. This is not the case of living salamanders where adult individuals present pedicellate monocuspid or bicuspid teeth on the premaxilla and maxilla, while different tooth rows could be present on the vomer. In extant and possibly most extinct archosaurs, teeth are generally thecodont and continuously replaced, with a thin enamel, dentine and cementum. They are mainly used for holding preys^30^, as it may be the case in Triassic temnospondyls. However, in anamniotes, the functional significance of plicidentine is still unclear but may related to increase the surface area for attachment tissues, to absorb shock stress during feeding, or both, as proposed for parareptiles^31^.

(c) Based on the discussed points above, the evolutionary ecology of the stereospondyls may be composed of different ecomorphotypes, with a relatively flattened skull and a snout morphology varying from a tube (in lonchorhynchine trematosaurs) to a broad and short spatula (in metoposaurs). In archosaurs, and neosuchians in particular, the flattening of the skull is related to the rostral shape, which shows two lateral morphotypes: platyrostral (flat-snouted) and oreinirostral (“hill-like snouted”)^23^,^32^. In stereospondyls and most of the temnospondyls, the lateral profile is only platyrostral and consequently less adapted to resist both bending and torsional forces. The fact that stereospondyls did not explore an oreinirostral morphotype probably limited their ecomorphological diversity, especially on land, and may be linked to the absence of a secondary palate and the presence of a cultriform process which did not support important stress levels during most of the feeding behaviours.

Giant amphibians were abundant and dominant after the end-Permian mass extinction. They occupied many vacant ecological niches in continental and marine ecosystems of the Triassic. Archosauromorphs also survived the end-Permian extinction and radiated during the Triassic. Early Triassic archosauriforms (as proterosuchids) occupied terrestrial ecosystems while their diversity increases during the Middle Triassic with small-sized meso- and top predators such as poposauroids and “rauisuchians”. Later, during the Late Triassic ornitosuchids, early dinosaurs and early crocodylomorphs occupied these ecosystems^33^,^34^. Some archosauromorph groups developed morphofunctional innovations such as a secondary palate, which appeared in Early Jurassic neosuchians^35^. This secondary palate may increase their biomechanical capabilities and may correspond to an ecological advantage, in term of natural selection, compared with stereospondyls living in the same ecosystems. This is also suggested by the co-occurrence of metoposaurian temnospondyls and pseudosuchian phytosaurs in many non-marine Late Triassic localities around the world^36^,^37^. Even if their respective ecological niches seem differentiated, archosaurs and giant stereospondyls therefore shared the same non-marine environments during the Late Triassic. The latter decreased in biodiversity from the Early Jurassic, while various archosaurian clades increased in continental ecosystems probably linked to the niche expansion of archosaurs thanks to these key morphofunctional innovations.

## Methods

### Institutional abbreviations

University of California Museum of Paleontology, USA: UCMP; University of California Museum of Vertebrate Zoology, USA: MZV; Museu de Ciències Naturals de Barcelona, Catalonia, Spain: MZB; Muséum national d’Histoire naturelle, France: MNHN.

Four skulls were scanned and modeled under a Finite Element Analysis (FEA):

-UCMP 57749; complete adult skull of the capitosaurian stereospondyl *Stanocephalosaurus birdi*, type species of the genus, from the Early Triassic of North America (the genus is also known in the Early-Middle Triassic of Africa and Asia^38^,^39^.

-MNHN-MSNM V2992; complete adult skull of the capitosaurian stereospondyl *Edingerella madagascariensis* from the Early Triassic of Madagascar (the species is also documented by an ontogenetical series^14^,^17^).

-MVZ 69449; complete adult skull of the living *Dicamptodon ensatus*, giant Pacific salamander (the genus includes four species, three with a terrestrial adult stage, reaching a total length of 350 mm, with large heads and robust jaws). *Dicamptodon ensatus* is capable of eating large preys (e.g. mices) relatively to their body size^40^.

-MZB 92-023; complete adult skull of the living *Alligator mississippiensis* (this archosaurian genus also includes the Chinese *A. sinensis*). *A. mississippiensis* reaches 4 meters long, and feeds on invertebrates and vertebrates (e.g. fishes, turtles, birds, mammals). This semiaquatic archosaurian represents a good model for biomechanics because many of its biological and anatomical aspects are well known (e.g., jaw adductor muscles, feeding functional morphology, ontogenetical diet shifts^41^,^42^).

### Computed tomography

The adult skulls of *S. birdi*, *E. madagascariensis* and *A. mississippiensis* were scanned at the Hospital Mútua de Terrassa (Catalonia, Spain) using a medical CT scanner Siemens Sensation 16. *S. birdi* was scanned at 140 kV and 300 mA, obtaining 0.449 mm of pixel size and an output of 512 × 512 pixels per slice with an interslice space of 0.3 mm. *E. madagascariensis* was scanned at 140 kV and 150 mA, obtaining 0.586 of pixel size and an output of 512 × 512 pixels per slice with an interslice space of 0.1 mm. *A. mississippiensis* was scanned at 120 kV and 200 mA, obtaining 0.586 mm of pixel size and an output of 512 × 512 pixels per slice with an interslice space of 0.5 mm. *Dicamptodon ensatus* (MVZ 69449) was scanned at the University of Texas, with the High-Resolution X-ray Computed Tomography Facility-Digital Morphology Group (www.digimorph.org), and using an ACTIS CT scanner. The skull of *D. ensatus* was scanned at 120 kV and 0.2 mA, obtaining 89 μm of voxel size and an output of 1,024 × 1,024 pixels per slice.

### Geometrical reconstruction

The CT data of each scanned model was imported to the software Avizo 7.0 (FEI-VSG company), where a reconstruction and segmentation was performed for the 3D models of *S. birdi, D. ensatus* and *A. mississippiensis* (Supplementary Figs S5 and S6). The digital 3D models were converted to a CAD format. The presence of irregularities in the surface of the reconstructed models is due to the quality of the CT scans and was repaired using refinement and smoothing tools.

In *E. madagascariensis*, additional refinements were required to obtain the final CAD model (Supplementary Fig. S6). Inner regions, as the internal area between the vomer and the skull roof bones, the endocranial region and the inner part of the cultriform process were reconstructed using a previous 3D model for this taxon^20^.

### Model properties

A Structural Static Analysis of each skull was performed using the Finite Element Package ANSYS 14.5 in a Dell Precision™ Workstation T7600 with 32 GB (4X8GB) and 1600 MHz.

The cranial bone properties for living salamanders or any other anamniote are unknown but are well known for different reptile groups. To facilitate comparisons between the models, elastic, lineal and homogeneous material properties were assumed using the following values: E (Young’s modulus): 6.65 GPa and m (Poisson’s ratio) 0.35 of Currey^43^. However, Gil *et al.*^44^ demonstrated that, in a comparative analysis of different FEA models, the latter value is not crucial. In this study, the interest of the comparison between the models raises in the Von Mises stress distribution. Bone can be assumed as brittle^45^ or ductile^46^ material. According to Doblare^45^, the Von Mises criterion is the most used and useful criterion for predicting the yield and fracture location in bone when isotropic material properties and ductile material is assumed in cortical bone.

The skulls were meshed with an adaptive mesh using hexahedral elements^47^ with the following parameters: *S. birdi* about 1.8 millions of elements and 2.6 millions of nodes*; E. madagascariensis* about 2.1 millions of elements and 2.9 Millions of nodes; *D. ensatus* about 3.2 millions of elements and 4.5 millions of nodes; and *A. mississippiensis* about 3 millions of elements and 4.4 millions of nodes (Supplementary Fig. S5).

### Boundary and loading conditions

The adductor musculature was modelled using the trigeminal topological paradigm^48^ as the criteria to identify muscle homology. The Adductor mandibulae internus (AMI), the Adductor mandibulae externus (AME) and the Adductor mandibulae posterior (AMP) were considered (Table 1, Supplementary Fig. S6). The musculoskeletal anatomical reconstruction in extinct taxa is particularly difficult because data are missing. The reconstruction of soft tissues in fossil taxa is mainly based on the attachments of the musculature preserved in bones, and requires to be inferred using a phylogenetic approach as the extant phylogenetic bracket^49^. Herein, we present first an overview of the adductor musculature for the living taxa analysed, in order to discuss the digital reconstruction of the adductor musculature in the extinct taxa analysed.

The AMI is divided into pseudotemporalis and pteygoideus among tetrapods^41^,^50^,^51^. In lissamphibian urodeles, it originates from the fasciae of the epaxialis musculature and more anteriorly from the parietal, frontal, and nasal regions. Its fibers run ventrally to insert slightly anterior to the insertion site of the AME on the mandible^52^. In crocodilians, the pseudotemporalis is less developed and herein not considered. The pterygoideus muscles occupy important regions of the head: this is particularly the case of the pterygoideus dorsalis, which occupies the dorsal surface of the palate and suborbital space, with attachments on the dorsal surface of the palatine, pterygoid, ectopterygoid, the ventral surface of the interorbital septum, the ventrolateral surface of the lacrimal, the dorsomedial surface of the maxilla/ectoptergyoid articulation, and the suborbital fenestra. This muscle also runs in different directions; caudally through the postnasal fenestra and attaches to the ventromedial surface of the angular and articular, ventrally through the jaw joint with the ventromedial edge of the medial mandibular fossa, and caudally through the pterygoid flange^41^,^53^.

The AME is large and undivided in the lissamphibian urodeles. It originates typically from the anterior part of the squamosal, runs ventrally and inserts on the coronoid part of the mandible^50^,^52^. In crocodilians, it is the most functionally muscle and is usually partitioned into various sections; superficial (M. adductor mandibulae externus superficialis), medial (M. adductor mandibulae externus medialis), and deep (M. adductor mandibulae externus profundus). It also occupies most of the temporal fossa and lateral region of the adductor chamber, and has broad attachments to the dorsotemporal fossa and medial surfaces of the laterally bounding dermocranium (postorbital, squamosal). AME origins on the ventrolateral surface of the parietal, the rostromedial surface of the quadrate, and the rostrolateral surface of the quadrate and quadratojugal^41^,^53^.

The AMP is a small and poorly differentiated muscle in lissamaphibian urodeles^50^,^54^. It is included with AME in the *Dicamptodon ensatus* model. In crocodilian archosaurs, the AMP represents one of the larger adductor muscles. It originates on the rostral surface of the quadrate, and inserts on the medial region of the mandible, occupying most of the medial mandibular fossa. In this fossa, the AMP attaches on the dorsal surface of the angular, the rostral surface of the articular, and the medial surface of the dermis, overlying the external mandibular fenestra^41^,^53^.

In early tetrapods, such as temnospondyls, attempts of reconstruction of the cranial musculature have been published^50^,^55^,^56^. In various temnospondyls (e.g., *Dendrerpeton*, plagiosaurids, stereospondyls), the Adductor Musculature Internus (AMI), Externus (AME) and Posterior (AMP) can be reconstructed thanks to well preserved fossil specimens showing insertion areas for the musculature: the insertion area of the AMI, reduced in some temnospondyls (incl. stereospondyls), is located on the ventral part of the postorbital bones nearing the orbit (postfrontal, postorbital and supratemporal)^50^,^56^ (Supplementary Fig. S6). The AME is inserted on the ventral part of the cheek region (squamosal) in stereospondyls, being more developed than that of the lissamphibian urodeles (Supplementary Fig. S6). The AME is placed adjacent to the AMP and this latter musculature is more developed than is found in lissamphibian urodeles^50^,^56^. Moreover, some temnospondyls (like plagiosaurids) present, in adult specimens, a well ossified hyobranchial skeleton probably performing a strong suction feeding^56^, while in other groups (like stereospondyls), the hyobranchial skeleton is unknown and probably was cartilaginous or reduced^4^.

Considering the described information on adductor musculature in living and extinct taxa, the muscular insertion areas of AMI, AME and AMP respectively were defined in the 3D geometric models in order to apply the forces of the muscular contraction during the prehension/bite (Supplementary Fig. S6).

Three loading cases were analysed considering a bilateral, unilateral and lateral prehension/bite. Lateral prehension simulation was not performed in *D. ensatus* because this behaviour has not been described in urodeles. The bilateral case simulates a bite on the left and right sides, while the unilateral case simulates a bite on the right side only. The lateral case simulates a lateral loading direction to generate a within-plane lateral bend to the snout. This simulates rapid sideways sweeps of the head through the water.

In *D. ensatus*, the prehension zone is located anteriorly, on the premaxilla-maxilla suture region, applying a fixed boundary condition. In *A. mississipiensis*, it is located at the level of the maxilla convexity, near the front of the jaws (upper caniniform teeth), where preys are initially captured^57^. In *S. birdi* and *E. madagascariensis*, it is located in the premaxilla-maxilla suture region. In all the cases, fixed boundary conditions in x, y and z directions were applied at the prehension zones. Displacements were fixed at the occipital condyles in the x-direction related with the vertebral column, and at the jaw joint areas in y-direction, where the skull is in contact with the lower jaw to avoid rigid motion.

### Scaling the models

To compare the performances of structures that differ in shape and size, the values of muscular contraction pressure were calculated according to the methodology developed by Marcé-Nogué *et al.*^58^ and rearranged for 3D models by Fortuny *et al.*^21^. This methodology relates the volume of each specimen with the muscular pressures applied by a 2/3 power relationship, and agrees with the allometric proportions of the species^59^. To scale the muscular force, a reference model is necessary. As the goal is to analyse the ecological role of Triassic temnospondyls, the two extant taxa *D. ensatus* and *A. mississipiensis* (Crocodylia) were used as reference models:

In this manner, both models were scaled with respect to volume, and using only the force values, as stated in equation (1), allowing the comparison between them.


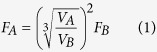


And finally, the muscular pressure (P) of both models A and B were related with the variation of the volume (V) of the skull as equation (2) states in function of the area of muscular insertion (MI).


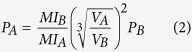


### Cases of study

Ten cases of study were analysed according to the equations proposed to scale the models (Table 1). The values for the muscular pressure of AMI, AME and AMP after applying the scaling method were also included in the Table 1. A constant PCSA was assumed for these muscles, applying the muscular pressure directly to the area of its muscular insertion (MI), and considering that all muscles work simultaneously and maximally. The direction of the muscular forces was obtained from the virtual line that joins the centroid of the insertion areas of the skull muscles with its respective centroid in the lower jaw. Regarding the reference taxa, for *A. mississippiensis,* the muscular contraction force values were obtained from Gignac^42^ and applied according the directions proposed by Porro *et al.*^60^. The values of the areas of muscular insertion (MI) of *A. mississippiensis* were obtained directly from the geometrical model. For the studied amphibians (*D. ensatus* and the Triassic temnospondyls), comparative data on the muscular values in salamanders and amphibians in general are missing. Herein, we follow Fortuny *et al.*^21^ for the analysis of the Chinese Giant salamander, *A. davidianus*. The force production/potential of individual salamander jaw muscles is unknown. The values of the MI areas in *D. ensatus* were obtained directly from the geometrical model and a value of 0.3 MPa (force per unit area) was used as a contractile pressure in each muscle. This value, estimated from Alexander^61^, is considered as arbitrary for an isometric contraction of each adductor muscle. The values of the MI areas in *D. ensatus* were also obtained directly from the geometrical model, whereas the direction of the muscular forces were obtained from the virtual line that joins the centroid of the insertion areas of the skull muscles with its respective centroid in the lower jaw. The values of *E. madagascariensis* and *S. birdi* were scaled from the reference models. As explained above, the values of the MI areas were reconstructed and obtained directly from the geometric models. The gape angle used is of 30° for *A. mississippiensis* (following Porro *et al.*^60^), 15° for *D. ensatus* (following Fortuny *et al.*^21^), and 30° for *E. madagascariensis* and *S. birdi* (but also tested under 15°; Supplementary Table S1).

For the bilateral and unilateral bites and according to these values of reference, the values for the muscular contraction in cases E, F, G, H, I and J (Table 1) were obtained using equation (2). As previously explained, in *D. ensatus*, the AME and AMP are joined in the same insertion area. In cases A, C and D (Table 1), the constant value of 0.3 MPa is also assumed for all the muscles.

For the lateral bite, 100 N were assumed as a reference value for the bite force in *A. mississippiensis.* The lateral bite was not considered for *D. ensatus*. However, in the cases of the temnospondyls have been loaded under a lateral biting using *D. ensatus* as a reference model, a reference value of 1 N force was used. This change in the value is due to the different sizes of the reference models to generate low stress values. The scaled force in cases F, G, H, I and J (Table 1) was obtained using equation (1).

### Multivariate Analysis

To analyse the response of the stress levels in different regions of the skull and in a multivariate approach, PCAs for each case and scale model were tested. These PCAs were developed using as variables the stress values recorded at different homologous points in each model, and using the variables of all taxa. A PCA can also be based on the variance-covariance matrix of the original variables or using the correlation matrix. This last case is applied whenever the variables have different scales or units. However, as we are analysing the variability of the stress values, and all the variables are in the same units, the variance-covariance option is here selected.

## Additional Information

**How to cite this article**: Fortuny, J. *et al.* Comparative 3D analyses and palaeoecology of giant early amphibians (Temnospondyli: Stereospondyli). *Sci. Rep.* **6**, 30387; doi: 10.1038/srep30387 (2016).

## Supplementary Material

Supplementary Video 1

Supplementary Video 2

Supplementary Video 3

Supplementary Information

## Figures and Tables

**Table 1:** Cases analysed in the present study.

CASE	Taxa	Scaling	Reference	AME [MPa]	AMI [MPa]	AMP [MPa]	F Lateral [N]
A	*Dicamptodon ensatus*	Non-Scaled	—	0.3	0.3	0.3*	1**
B	*Alligator mississippiensis*	Non-scaled	—	AMES = 0.0369 AMEM = 0.0334 AMEP = 0.0651	0.2772	0.0689	100
C	*Edingerella madagascariensis*	Non-scaled	—	0.3	0.3	0.3	100
D	*Stanocephalosaurus birdi*	Non-scaled	—	0.3	0.3	0.3	100
E	*Dicamptodon ensatus*	Scaled	*Alligator mississippiensis*	0.0336	0.0226	0.0336*	1.1035
F	*Alligator mississippiensis*	Scaled	*Dicamptodon ensatus*	AMES = 0.4678 AMEM = 1.7747 AMEP = 4.5166	3.6743	0.6577	90.615
G	*Edingerella madagascariensis*	Scaled	*Alligator mississippiensis*	0.02325	0.3787	0.09484	21.33
H	*Edingerella madagascariensis*	Scaled	*Dicamptodon ensatus*	0.1686	5.0186	0.1686	19.4
I	*Stanocephalosaurus birdi*	Scaled	*Alligator mississippiensis*	0.0105	0.1596	0.0281	34.88
J	*Stanocephalosaurus birdi*	Scaled	*Dicamptodon ensatus*	0.0698	2.1160	0.0698	31.60

Muscle forces applied under non-scaled and volume scaled cases.

^*^In *D. ensatus*, AME and AMP were considered in the same insertion area.

^**^Lateral biting was not considered in *D. ensatus.* In *A. mississippiensis,* AME was composed by M. adductor mandibulae externus superficialis (AMES), M. adductor mandibulae externus medialis (AMEM), and M. adductor mandibulae externus profundus (AMEP).
