# Supplementary material for: Comparative 3D analyses and palaeoecology of giant early amphibians (Temnospondyli: Stereospondyli)
Source: Sci Rep. 2016 Jul 26;6:30387. doi: 10.1038/srep30387 (PMC4960601; doi:10.1038/srep30387)
Supplement: Supplementary Information [file srep30387-s4.pdf]

Comparative 3D analyses and palaeoecology of giant early amphibians (Temnospondyli: Stereospondyli)

Josep Fortuny*, Jordi Marcé-Nogué, J.-Sébastien Steyer, Soledad de Esteban-Trivigno, Eudald Mujal, Lluís Gil

*corresponding author: josep.fortuny@icp.cat

Supplementary Information

Supplementary Data

Supplementary Tables S1, S2, S3, S4, S5

Supplementary Figures S1, S2, S3, S4, S5, S6

Supplementary Videos S1, S2, S3

Supplementary Data

Extant taxa

Lissamphibia

The adult skull model of *Dicamptodon ensatus* shows under a bilateral loading that maximum stress peaks around the centre of the frontals, affecting also the posterior part of the prefrontals but more importantly the vomer-parapshenoid suture (Fig. 1). Stresses are also present on the posterior part of the skull including the squamosal and quadrates and, in a lesser way, in the parietals and exoccipitals. The palate of *D. ensatus* reveals that, under this feeding behaviour, the anterior part of the skull present moderate-high stresses on the vomer-parasphenoid suture, the posterior margin of the vomerine plate, and on the posterior part of the maxilla. The posterior part of the parasphenoid shows low levels of stress, while no stress is present on the pterygoid.

Regarding the unilateral behaviour (Fig. 2), the lissamphibian skull present similar results to the bilateral case, but mainly increased in all the regions: the stress on the center of the frontals focuses on the frontal-prefrontal margin, while on the posterior part of the skull, the general pattern is similar to the bilateral loading but increases in the posterior part of the parasphenoid, exoccipitals and quadrates.

Crocodylia

Under a bilateral loading, the skull of *Alligator mississippiensis* reveals any or low levels of stress around the snout. The stress level increases around the preorbital region, including the posterior part of the maxilla, lacrimal, prefrontal and nasal (Fig. 1). These stress regions forms a continuous belt into the palate portion of the maxilla and palatine. Few stress regions are present around the orbit, while moderate stress levels are present in the posterior part of the skull, including the parietals and especially the

supraoccipitals. No stress or very low levels are found on the pterygoids and ectopterygoids, while moderate stress levels are present on the exoccipitals, basioccipitals and basisphenoids.

A unilateral feeding behaviour in *A. mississippiensis* reveals a similar pattern to the bilateral feeding case (Fig. 2); with maximum stress peaking around the posterior part of the lacrimal, maxilla and prefrontal, continuing to the palatine and maxilla part of the palate, and around the quadrates and quadratojugals.

Finally, under a lateral strike (Fig. 3), considering hunting prey by using a rapid sideways sweep of the head, the *Alligator* model reveals very low or any stress on the anterior part of the skull, especially on the skull roof (premaxilla, maxilla, nasals), while low levels are present on the secondary palate region (especially on the maxilla) (Fig. 3). Stresses increases on the cheek region (jugals) and the posterior part of the skull (squamosal, quadrate), increasing on the occipital condyle area, while, in palatal view, stresses are low, especially on the pterygoid.

Supplementary Tables

Table S1. Estimated bite forces - as reaction forces - in the bilateral and unilateral case.

Table S2. Von Mises Stress values at several homologous points of the 3D skull models under bilateral loading.

Table S3. Von Mises Stress values at several homologous points of the 3D skull models under unilateral loading.

Table S4. Von Mises Stress values at several homologous points of the 3D skull models under lateral loading.

Table S5. Principal Component Analyses of the different cases: percent of variance explained and loadings for the different variables.

Table S1. Estimated bite forces - as reaction forces - in the bilateral and unilateral case. Lateral case not available because the bite force was an input parameter of the model, not a boundary condition.

Taxa	Scaling	Reference	Bite force in one teeth [N]			
			BILATERAL BITE 15º	BILATERAL BITE 30º	UNILATERAL BITE 15º	UNILATERAL BITE 30º
Dicamptodon ensatus	Non-Scaled	-	3.15		25.19	
Alligator mississippiensis	Non-scaled	-		82.26		454.07
Edingerella madagascariensis	Non-scaled	-	42.75	32.02	479.14	317.99
Stanocephalosaurus birdi	Non-scaled	-	130.31	135.84	1899	1215.3
Edingerella madagascariensis	Scaled	*Alligator mississippiensis*	6.963	7.8918	95.5	65.03
Edingerella madagascariensis	Scaled	*Dicamptodon ensatus*	70.77	86.84	701.2	506
Stanocephalosaurus birdi	Scaled	*Alligator mississippiensis*	9.569	19.697	153.42	102.12
Stanocephalosaurus birdi	Scaled	*Dicamptodon ensatus*	115.45	236.04	1131.8	815.95

Table S2. Von Mises Stress values at several homologous points of the 3D skull models under bilateral loading. PPH: centre of the Parasphenoid, C: most external point of the occipital condyle, Pt: centre of the posterior margin of the pterygoid, V: middle centered point of the vomer, Premx: most anterior point between the premaxilla, IO: centre of the interorbital region, NS: middle point between the nasal sutures.

CASE	Taxa	Scaling	Reference	PPH	C	Pt	V	Premx	IO	NS
A	*Dicamptodon ensatus*	Non-Scaled	-	1.8347	5.9782	4.7294	4.3115	0.2329	4.9611	1.7205
B	*Alligator mississippiensis*	Non-scaled	-		1.7599	0.5307		0.1267	0.1613	0.0423
C	*Edingerella madagascariensis*	Non-scaled	-	2.6646	0.9181	1.5427	0.1111	0.4648	1.1725	0.1705
D	*Stanocephalosaurus birdi*	Non-scaled	-	5.2905	5.7193	1.6105	2.9016	1.6724	3.5545	4.0663
E	*Dicamptodon ensatus*	Scaled	*Alligator mississippiensis*	0.2084	0.0475	0.3009	0.9848	0.8431	0.2180	0.1028
F	*Alligator mississippiensis*	Scaled	*Dicamptodon ensatus*		14.9740	5.6176		0.1236	3.0831	0.5071
G	*Edingerella madagascariensis*	Scaled	*Alligator mississippiensis*	0.6788	0.1455	0.2306	0.0422	0.0038	0.6519	0.0103
H	*Edingerella madagascariensis*	Scaled	*Dicamptodon ensatus*	5.9601	0.5887	3.7201	0.7229	0.0849	8.1269	0.3270
I	*Stanocephalosaurus birdi*	Scaled	*Alligator mississippiensis*	0.4106	0.8859	0.1842	0.1514	0.1201	1.3281	0.2826

J	*Stanocephalosaurus birdi*	Scaled	*Dicamptodon ensatus*	4.5287	8.5227	1.0263	0.8853	1.1871	17.3320	2.8418

Table S3. Von Mises Stress values at several homologous points of the 3D skull models under unilateral loading. PPH: centre of the Parasphenoid, C: most external point of the occipital condyle, Pt: centre of the posterior margin of the pterygoid, V: middle centered point of the vomer, Premx: most anterior point between the premaxilla, IO: center of the interorbital region, NS: middle point between the nasal sutures.

CASE	Taxa	Scaling	Reference	PPH	C	Pt	V	Premx	IO	NS
A	*Dicamptodon ensatus*	Non-Scaled	-	7.2279	3.3321	3.9164	9.8979	12.4360	6.4929	8.3742
B	*Alligator mississippiensis*	Non-scaled	-		5.4035	0.7593		0.1035	0.3880	0.0742
C	*Edingerella madagascariensis*	Non-scaled	-	11.1440	20.1450	4.8442	3.0968	0.2687	2.3938	1.7612
D	*Stanocephalosaurus birdi*	Non-scaled	-	45.0330	83.3000	4.8775	5.7845	10.1640	8.4929	11.9340
E	*Dicamptodon ensatus*	Scaled	*Alligator mississippiensis*	0.2440	0.2532	0.2487	0.3621	0.0193	0.3963	0.1528
F	*Alligator mississippiensis*	Scaled	*Dicamptodon ensatus*		148.2800	13.7170		1.6967	8.5523	3.9084
G	*Edingerella madagascariensis*	Scaled	*Alligator mississippiensis*	2.1044	3.2130	1.0380	0.6950	0.1195	0.7674	0.3856
H	*Edingerella madagascariensis*	Scaled	*Dicamptodon ensatus*	15.3130	24.5860	2.8478	5.7129	1.8899	8.0280	3.6725
I	*Stanocephalosaurus birdi*	Scaled	*Alligator mississippiensis*	3.7142	7.2547	0.4027	0.4211	0.9787	0.4976	1.0622

J	*Stanocephalosaurus birdi*	Scaled	*Dicamptodon ensatus*	24.0610	51.5870	2.7383	3.3471	8.0997	5.2833	8.8988

Table S4. Von Mises Stress values at several homologous points of the 3D skull models under lateral loading. PPH: center of the Parasphenoid, C: most external point of the occipital condyle, Pt: center of the posterior margin of the pterygoid, V: middle centered point of the vomer, Premx: most anterior point between the premaxilla, IO: centre of the interorbital region, NS: middle point between the nasal sutures.

CASE	Taxa	Scaling	Reference	PPH	C	Pt	V	Premx	IO	NS
A	*Dicamptodon ensatus*	Non-Scaled	-	-	-	-	-	-	-	-
B	*Alligator mississippiensis*	Non-scaled	-		18.8390	0.2370		0.2109	0.7450	0.3947
C	*Edingerella madagascariensis*	Non-scaled	-	7.1898	13.9070	3.4441	1.7975	0.3388	0.7829	1.3397
D	*Stanocephalosaurus birdi*	Non-scaled	-	5.3132	10.9310	1.0890	2.2225	0.3943	1.0055	0.9797
E	*Dicamptodon ensatus*	Scaled	*Alligator mississippiensis*	-	-	-	-	-	-	-
F	*Alligator mississippiensis*	Scaled	*Dicamptodon ensatus*		11.1240	0.4007		0.2101	0.7142	0.2396
G	*Edingerella madagascariensis*	Scaled	*Alligator mississippiensis*	1.5288	2.8769	0.7425	0.9135	0.0708	0.1671	0.2868
H	*Edingerella madagascariensis*	Scaled	*Dicamptodon ensatus*	143.4000	1696.9900	70.6630	61.6980	8.9242	16.1630	23.7560
I	*Stanocephalosaurus birdi*	Scaled	*Alligator mississippiensis*	1.5274	3.7724	0.3943	0.8352	0.1334	0.3505	0.3979

J	*Stanocephalosaurus birdi*	Scaled	*Dicamptodon ensatus*	2.0294	3.3571	0.3544	0.8608	0.1393	0.3156	0.3191

Table S5. Principal Component Analyses of the different cases: percent of variance explained and loadings for the different variables. For the lateral case, *Dicamptodon* was not included in the analyses.

	Bilateral case scaled *Alligator*		Bilateral case scaled *Dicamptodon*		Unilateral case scaled *Alligator*	Unilateral case scaled *Dicamptodon*	Lateral case scaled *Alligator*	Lateral case scaled *Dicamptodon*
	PC 1	PC 2	PC 1	PC 2	PC1	**PC1**	PC1	PC1
Variance explained	61.2 %	31.8 %	61.3 %	38.1 %	96.4 %	99.3 %	99.9 %	100 %
Loadings for each variable:								
Pt: Pterygoid	0.13247	-0.18154	-0.26370	-0.12608	0.01886	0.075873	-0.02227	0.04155
Premx: Premaxilla	-0.23571	-0.39034	0.05226	0.06434	0.11542	-0.050139	0.00716	0.00517
IO:InterOrbital	-0.05462	0.89191	0.81335	0.47653	0.00069	0.011825	0.03176	0.00925
NS:Nasal suture	0.00298	0.13697	0.09821	0.11574	0.09792	-0.021108	0.00367	0.01388
C: Condyle	0.96120	-0.02044	-0.50650	0.85994	0.98830	0.99556	0.99922	0.99898

Supplementary Figures

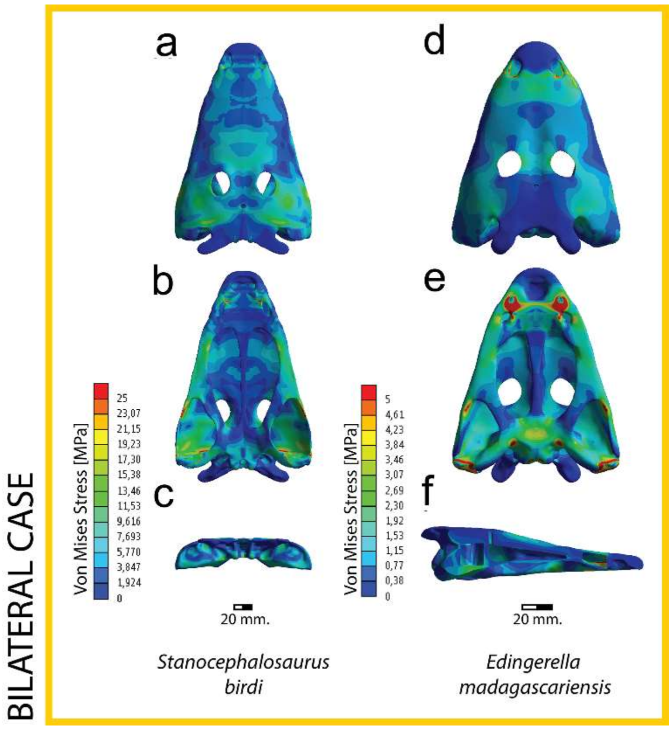

Figure S1. Von Mises stress results in MPa of bilateral biting in non-scaled models. (a-c), *Stanocephalosaurus birdi*; (d-f), *Edingerella madagascariensis*.

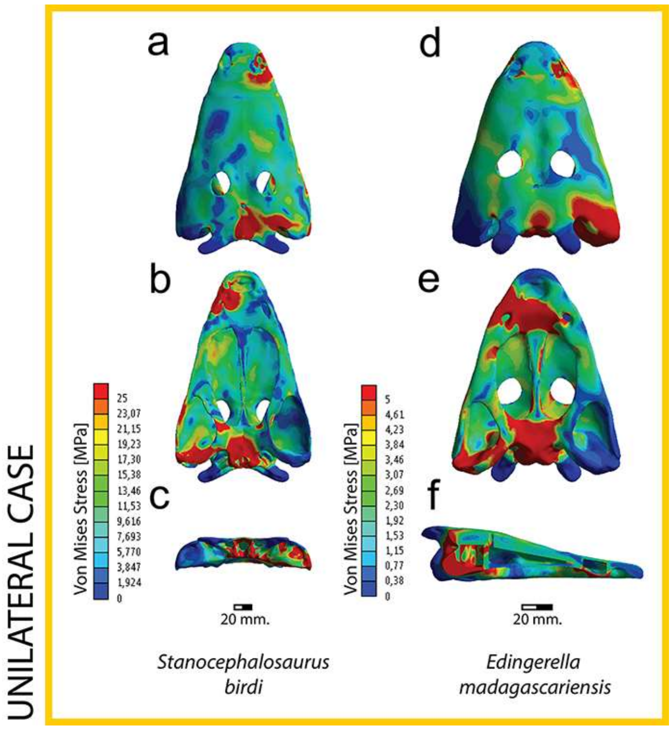

Figure S2. Von Mises stress results in MPa of unilateral biting in non-scaled models. (a-c), *Stanocephalosaurus birdi*; (d-f), *Edingerella madagascariensis*.

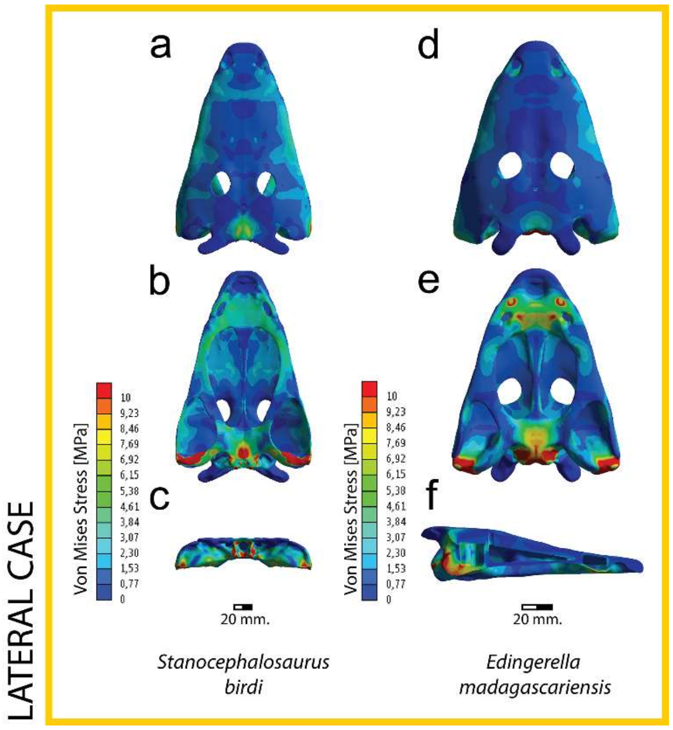

Figure S3. Von Mises stress results in MPa of lateral biting in non-scaled models. (a-c), *Stanocephalosaurus birdi*; (d-f) *Edingerella madagascariensis*.

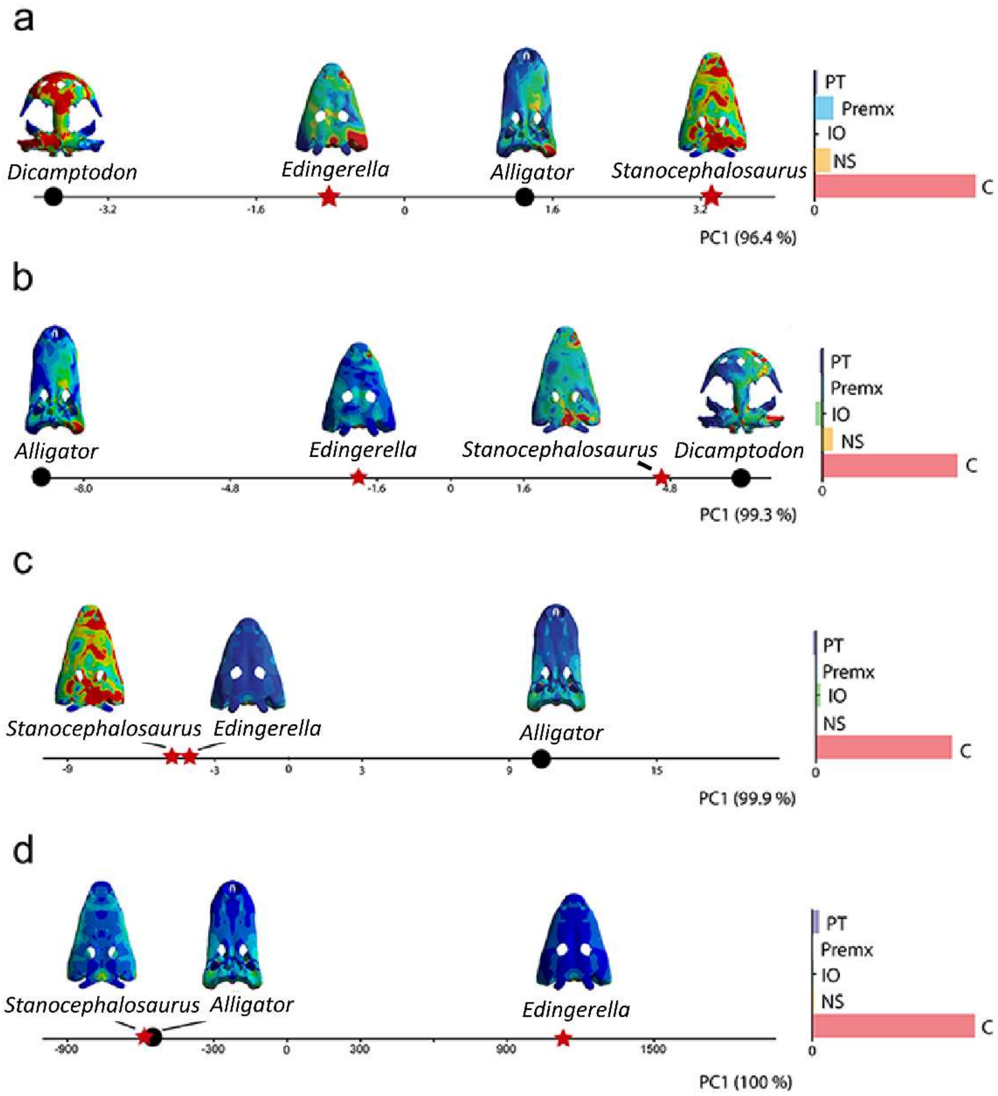

Figure S4. First Principal Component of PCAs. (a), Unilateral case scaled under *A. mississippiensis* reference model; (b), Unilateral case scaled under *D. ensatus* reference model; (c), Lateral case scaled under *Alligator* reference model; (d), Lateral case scaled under *D. ensatus* reference model. The bar graphs represent the loadings of the variables for each PC. Although all are represented at the same scale, for visualization purposes they have a different scale than the PCs dispersion graph. Percent of variance explained by each PC between brackets. Black circles correspond to the living taxa and red stars to extinct taxa. Abbreviations: C: most external point of the occipital condyle, Pt: centre of the posterior margin of the pterygoid, Premx: most anterior point between the

premaxilla, IO: centre of the interorbital region, NS: middle point between the nasal sutures.

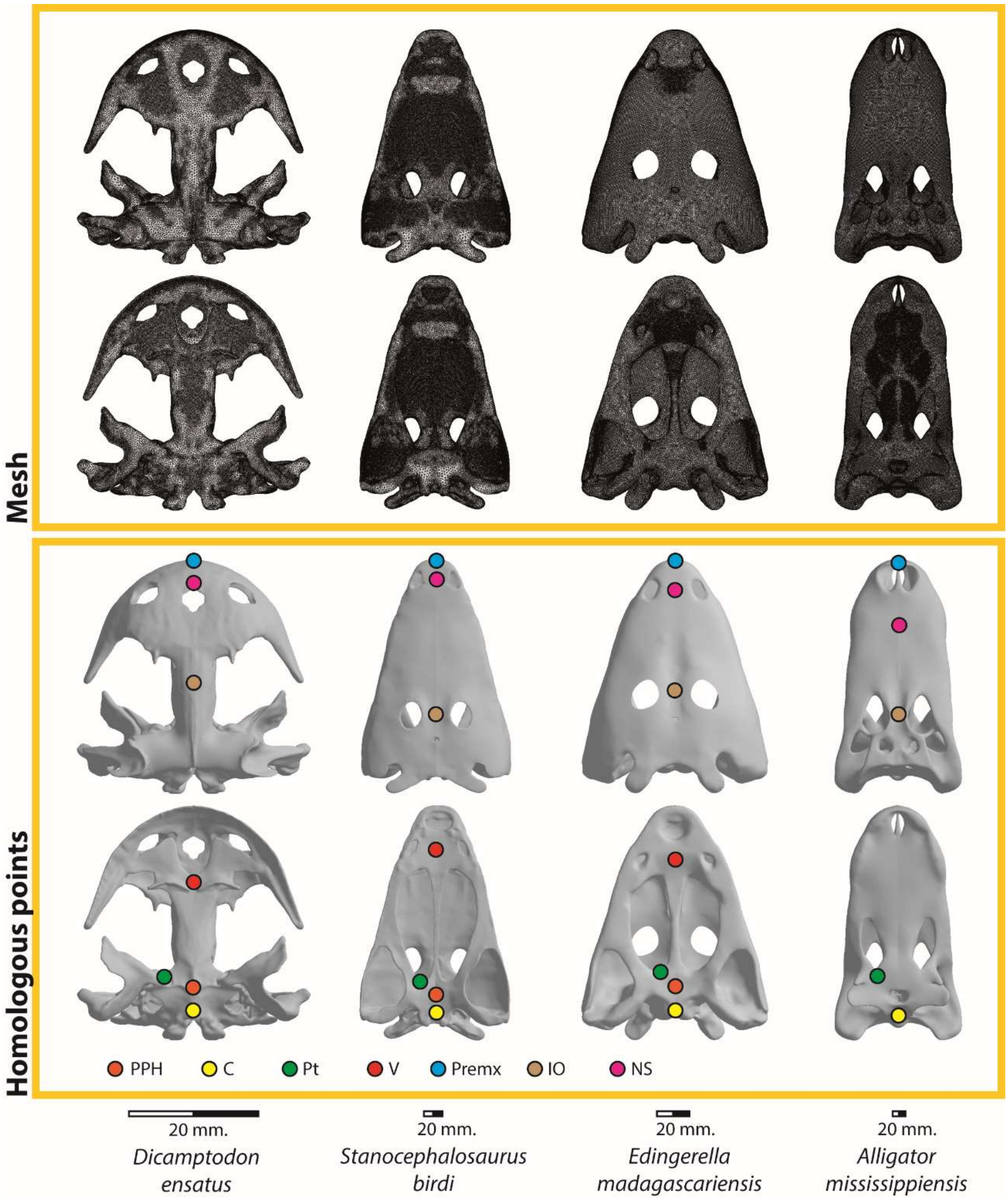

Figure S5. Meshed models with an adaptive mesh of hexahedral elements and homologous points used for the stress value measurements.

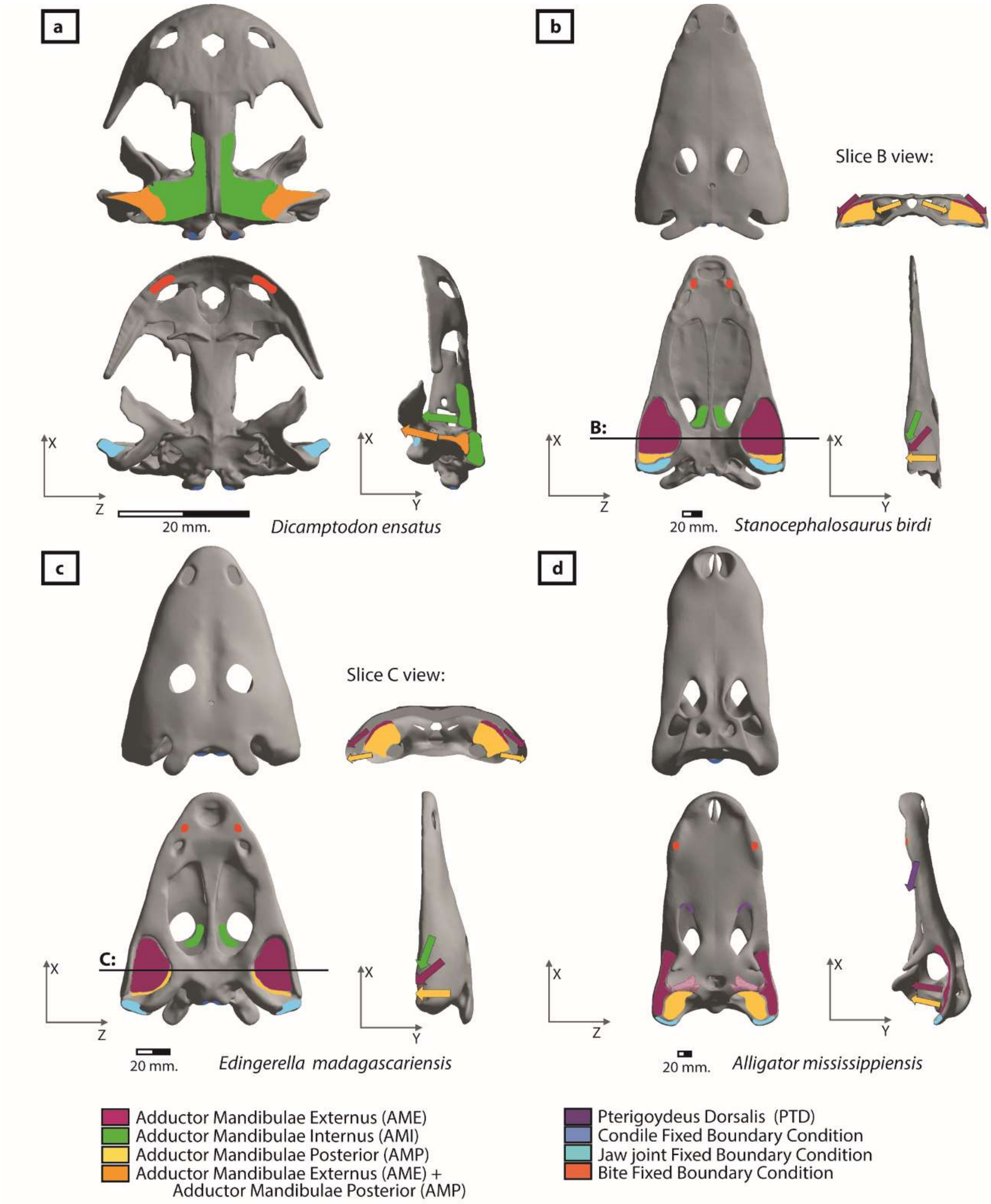

Figure S6. Boundary conditions of the four 3D skull models showing adductor musculature, biting and fixing points.

Supplementary Video Legends

Video S1. Bilateral biting in the analysed taxa. *S. birdi* scaled under *D.ensatus* reference model and *E. madagascariensis* under *A mississippiensis* reference model.

Video S2. Unilateral biting in the analysed taxa. *S. birdi* scaled under *D. ensatus* reference model and *E. madagascariensis* under *A. mississippiensis* reference model.

Video S3. Lateral biting in the analysed taxa. Both Triassic temnospondyls scaled under *A. mississippiensis* reference model.
